# Preparturient Oral Selenitetriglycerides Supplementation Elevates Erythrocyte Glutathione Peroxidase Activity and Modulates Hepatic *TNF-α*, *PPAR-α*, and *PPAR-δ* mRNA in Postparturient Holstein–Friesian Cows

**DOI:** 10.3390/ijms26168018

**Published:** 2025-08-19

**Authors:** Katarzyna Żarczyńska, Katarzyna Różańska, Paweł Brym, Dawid Tobolski

**Affiliations:** 1 Department and Clinic of Internal Diseases, Faculty of Veterinary Medicine, University of Warmia and Mazury in Olsztyn, 10-719 Olsztyn, Poland; katarzyna.zarczynska@uwm.edu.pl (K.Ż.);; 2 Department of Animal Genetics, Faculty of Animal Bioengineering, University of Warmia and Mazury in Olsztyn, 10-719 Olsztyn, Poland; pawbrym@uwm.edu.pl; 3 Department of Large Animal Diseases and Clinic, Institute of Veterinary Medicine, Warsaw University of Life Sciences, 02-787 Warsaw, Poland

**Keywords:** Holstein–Friesian cows, selenium supplementation, selenitetriglycerides, antioxidant enzymes, inflammation, metabolic regulation, transition period, periparturient health

## Abstract

The transition period in dairy cows, spanning late pregnancy and early lactation, is associated with substantial metabolic and immunological challenges, leading to increased oxidative stress and inflammation. Selenium (Se), particularly in organic forms, supports antioxidant defenses, immune function, and metabolic regulation. This study investigated the effects of supplementing periparturient Holstein–Friesian cows with orally administered selenitetriglycerides (0.5 mg Se/kg body weight/day starting 12 days before the expected calving date and continuing until parturition) on antioxidant enzyme activity and on the hepatic expression of key inflammatory and metabolic genes. Serum selenium concentrations and erythrocyte glutathione peroxidase (GSH-Px) activity were assessed before and after parturition, and hepatic gene expression levels of tumor necrosis factor alpha (*TNF-α*), peroxisome proliferator-activated receptor alpha (*PPAR-α*) and delta (*PPAR-δ*) were assessed 24 h and 7 days postpartum. Supplemented cows showed significantly elevated serum Se levels and increased GSH-Px activity, reflecting improved antioxidant capacity. Moreover, hepatic expression of *TNF-α* and *PPAR-δ* was significantly reduced postpartum in the supplemented group, whereas *PPAR-α* expression remained stable. These findings indicate that selenitetriglycerides effectively enhance antioxidant defenses, moderate inflammation, and stabilize metabolic pathways during the periparturient phase, potentially reducing postpartum metabolic disorders and improving dairy-cow health.

## 1. Introduction

Dairy cows experience profound metabolic and immune challenges during the transition from late gestation to early lactation. High genetic merit, selective breeding, and demanding production goals mean that these animals often encounter a negative energy balance (NEB) around parturition, demanding extensive mobilization of adipose stores [1,2,3]. This rapid lipolysis elevates circulating NEFA, fuels gluconeogenesis and ketogenesis, and can result in metabolic stress if regulatory pathways become overloaded [2,4]. Concurrently, endocrine changes and tissue remodeling in late pregnancy add further burdens, creating an environment where immune competence may be compromised and inflammation more easily triggered. The intersection of inflammatory cascades with metabolic adaptations is thus highly relevant to disorders such as ketosis, fatty liver, retained placenta, metritis, and reduced fertility [2,4].

Among the primary regulators of immune and metabolic integration, tumor necrosis factor alpha (TNF-α) is a key pro-inflammatory cytokine that is closely associated with metabolic dysregulation. Produced by immune cells and adipocytes, TNF-α exerts significant effects on insulin action, hepatic lipid flux, and overall energy homeostasis [5]. In states of overproduction, TNF-α can induce insulin resistance and amplify lipolysis, contributing to NEB [6]. Its upregulation in conditions like mastitis or severe hepatic steatosis highlights the frequent overlap between inflammation and metabolic stress in dairy cows [7,8]. Experimental administration of recombinant bovine TNF-α (rbTNF) to lactating cows has been shown to increase the hepatic triglyceride content [6]. Furthermore, elevated serum TNF activity has been observed in dairy cows affected with a naturally occurring fatty liver and is correlated with insulin resistance [7]. Studies have also indicated that rbTNF administration in early lactation can alter inflammatory responses and impair milk production and health [5,8]. Paradoxically, while moderate TNF-α activity is necessary for normal immune responses, chronic elevation can negatively impact postpartum recovery and metabolic health [5,6,7].

Peroxisome proliferator-activated receptors (PPARs) are ligand-activated transcription factors that orchestrate lipid metabolism, inflammation, and cellular differentiation. Although PPAR gamma has often been linked to adipogenesis, PPAR alpha (PPAR-α) and PPAR delta (PPAR-δ) also play key roles in dairy cattle. PPAR-α is found predominantly in the liver, where it governs fatty acid oxidation and ketogenesis. In early lactation, large amounts of NEFA enter hepatocytes for oxidation or ketone formation; this shift in metabolic substrate usage relies on PPAR-α-driven transcription [9]. Under ideal circumstances, PPAR-α helps to conserve glucose for milk production and prevents undue triglyceride accumulation in the liver. However, heightened TNF signaling can suppress PPAR-α expression, hindering NEFA oxidation and favoring a fatty liver or ketosis. Conversely, nutritional approaches that activate PPAR-α—such as specific fatty acid supplementation—may support hepatic oxidation and bolster metabolic adaptability [10].

PPAR-δ (also referred to as PPAR beta/delta) is more ubiquitously expressed. It regulates fatty acid oxidation in muscles, contributes to insulin sensitivity, and modulates inflammatory pathways in several cell types [11]. Its activation channels energy usage toward fat oxidation, thereby potentially mitigating insulin resistance and preserving glucose. Although direct in vivo data for dairy cows remain limited, in vitro findings suggest that PPAR-δ may be essential for embryo development and uterine receptivity, responding to prostaglandin I2 signals in related species [12]. Hence, appropriate coordination between PPAR-α and PPAR-δ is critical for balancing lipolysis, gluconeogenesis, and immune signals, especially when the periparturient timeframe intensifies metabolic demands.

Selenium (Se) is a trace element recognized for its antioxidant, anti-inflammatory, and immunomodulatory properties, intersecting with TNF-α and PPARs. Adequate Se enables the proper function of selenoproteins, notably those in the glutathione peroxidase family, which mitigate reactive oxygen species (ROS) and reduce tissue oxidative injury [13]. Se deficiency can trigger excess inflammation by amplifying the NF-κB/MAPK signaling pathway and, in turn, TNF expression [14]. Se supplementation, particularly via organic formulations, can moderate pro-inflammatory cascades, helping to preserve insulin responsiveness and limit metabolic disruption [15,16]. Additional studies show that Se helps to combat oxidative stress in multiple tissues, including mammary and uterine tissues, and can reduce postpartum disorders [17,18]. Intriguingly, some data imply that Se also modulates PPAR activity by promoting an intracellular redox balance that lessens NF-κB-induced cytokine release and supports selenoprotein-mediated detoxification [17]. This scenario maintains conditions favorable for PPAR-α-driven fatty acid oxidation, while the synergy with PPAR-δ may improve metabolic versatility and protect embryo development from oxidative damage [18]. Nevertheless, over-supplementation of Se can become deleterious, calling for precise dosing strategies to optimize antioxidant outcomes without incurring toxicity. The use of selenitetriglycerides reduces the risk of toxicity [13].

Taken together, TNF, PPAR-α, PPAR-δ, and selenium appear to be interwoven in a broader immunometabolic network. Excess TNF may hamper PPAR-based lipid oxidation, provoking hepatic fat accumulation and insulin resistance [19]. Adequate Se restrains the cytokine upsurge, thus allowing PPAR pathways to function optimally [17,20]. Where Se levels are low or PPAR signaling is undermined, transition cows are more prone to conditions like retained placenta, metritis, displaced abomasum, or ovarian cysts [15]. Farm-level interventions that target these molecular mechanisms—via dietary fatty acids, mineral fortification, or potential breeding for robust PPAR function—can alleviate postpartum complications and improve fertility. For example, certain polyunsaturated fatty acids serve as natural PPAR ligands, while a balanced Se intake can keep the immune system from being overwhelmed by oxidative or inflammatory pressures [21,22].

Building on previous insights, we examined how orally administered selenitetriglycerides influence TNF and PPAR signaling during the postpartum period in dairy cows. We hypothesized that supplementation with selenitetriglycerides would downregulate hepatic TNF and PPAR-δ expression while sparing PPAR-α, thus offering protective metabolic and immunological advantages in the early postpartum period.

## 2. Results

### 2.1. Biochemical Parameters

These biochemical data were partly reported in our previous publication [13] but are briefly restated here for the sake of completeness regarding selenium context. At the initial sampling, serum selenium concentrations in the control (CON) and experimental (STG) groups were very similar, at 26.82 µg/L (SD 6.02) and 28.17 µg/L (SD 6.41), respectively. In the CON group, selenium concentrations remained largely unchanged, with a slight increase on day 7 postpartum to 35.96 µg/L (SD 7.02). In contrast, STG-group cows given selenitetriglycerides displayed a marked rise at the second sampling (day −3 prepartum), reaching 352.25 µg/L (SD 112.59). Although this level declined thereafter, it remained significantly elevated (114.03 µg/L, SD 20.81) at day 7 postpartum relative to CON.

### 2.2. Antioxidant Parameters

**Figure 1:**
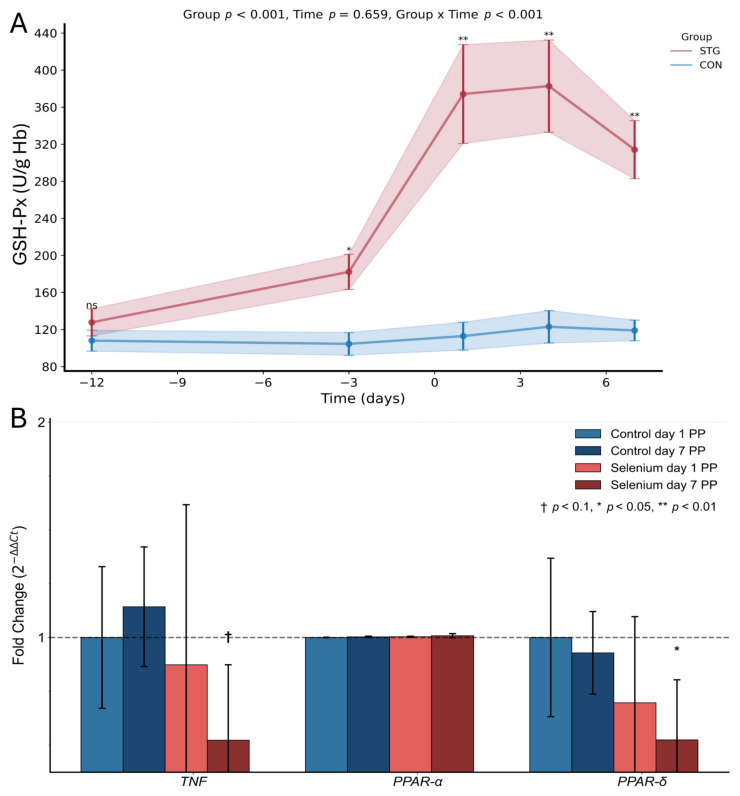
Effect of selenium supplementation on GSH-Px activity and gene expression in bovine liver. Panel (**A**) shows the dynamics of glutathione peroxidase (GSH-Px) activity measured in U/g Hb over time (−12 to 6 days), comparing selenium-treated group (STG, red line) with control group (CON, blue line). Statistical analysis indicates significant effects for group (*p* < 0.001) and group × time interaction (*p* < 0.001), but not for time alone (*p* = 0.659). Shaded areas represent standard error. Panel (**B**) presents the fold change in gene expression of *TNF*, *PPAR-α*, and *PPAR-δ* in bovine tissues collected on days 1 and 7 postpartum. The figure compares gene expression between control and selenium-supplemented groups. Bars represent the mean fold change in gene expression calculated using the 2^−ΔΔCt^ method, with error bars showing standard error (SE). The x-axis categorizes the three genes of interest (*TNF*, *PPAR-α*, and *PPAR-δ*), with separate bars for control day 1 PP (light blue), control day 7 PP (dark blue), selenium day 1 PP (light red), and selenium day 7 PP (dark red). The y-axis measures the fold change in gene expression relative to the control day 1 PP group (represented by the horizontal dashed line at fold change = 1). Statistical significance is denoted on the chart in comparison to control day 1 PP using specific symbols based on *p*-value thresholds: † *p* < 0.1 indicates a trend toward significance, * *p* < 0.05 indicates a statistically significant difference, ** *p* < 0.01 denotes a highly significant difference.

Glutathione peroxidase activity in whole blood of the examined cows at the first sampling was similar, measuring 108.01 U/gHb (SD 27.80) in the CON group and 127.76 U/gHb (SD 35.79) in the STG group (*p* = 0.873). In the non-supplemented cow group, the activity of this enzyme slightly decreased before calving to 104.52 U/gHb (SD 30.01) and then increased, reaching 119.03 U/gHb (SD 27.35) on the last day of the experiment (Figure 1A). In the STG-group cows, during the experiment, there was a significant increase in the activity of this enzyme compared to the first sampling and compared to cows from the CON group. The highest GSH-Px activity was observed on day 4 postpartum—382.75 U/gHb (SD 121.91). On the last day of the experiment, GSH-Px activity decreased and amounted to 314.15 U/gHb (SD 76.83). Except for the initial sampling on day -12, the differences in GSH-Px activity between the STG and CON groups were highly significant (*p* < 0.01) at all subsequent measurement points (Figure 1A).

### 2.3. Gene Expression of TNF, PPAR-α, and PPAR-δ

TNF levels did not differ significantly in the control group between day 1 and day 7 postpartum. By contrast, in cows receiving selenitetriglycerides, TNF exhibited a downward trend from day 1 to day 7 postpartum (fold change ~0.87 to 0.52, *p* = 0.065, †). Comparing the two groups at day 7 postpartum revealed that TNF was significantly lower in the selenium-treated group than in the control group (*p* = 0.026, *) (Figure 1B).

No notable variations in PPAR-α were detected in either group, with fold changes remaining close to unity and *p*-values exceeding 0.1. PPAR-δ also appeared stable in the control animals, yet in the supplemented group it decreased significantly by day 7 postpartum (fold change ~0.70 to 0.52, *p* = 0.041, *). Between-group analyses at day 7 postpartum confirmed a lower PPAR-δ expression in the STG-group cows compared with the controls (*p* = 0.041, *) (Figure 1B).

## 3. Discussion

Dairy cows, particularly those of high genetic merit, encounter a range of metabolic and immunological challenges during the transition period, which spans the final weeks of gestation and the initial stages of lactation. This interval is characterized by dramatic physiological shifts, including a marked NEB as the animal mobilizes body reserves to sustain milk production. In addition, endocrine variations, inflammatory processes, and transformations in nutrient partitioning are amplified during this timeframe, contributing to an elevated risk of conditions such as mastitis, metritis, ketosis, and hepatic steatosis [2,4,7]. These disorders, in turn, impair both productivity and reproductive performance. Because of their extensive interplay with immune, endocrine, and metabolic signaling networks, trace minerals—especially selenium (Se)—have received significant scientific attention. The study of selenium’s role in antioxidant defenses, immune modulation, and metabolic fine-tuning has expanded, resulting in new strategies for supplementation, such as the use of selenitetriglycerides, to support transition dairy cows [13,14,15]. Here, we provide an extensive discussion based on our findings and the existing literature, elucidating how selenitetriglycerides can address oxidative stress and inflammatory cascades, help to stabilize hepatic and adipose tissue metabolism, and potentially enhance postpartum recovery and fertility in high-yielding cows.

A growing body of evidence highlights selenium as a fundamental micronutrient that supports a variety of selenoproteins, most notably glutathione peroxidase (GSH-Px) and thioredoxin reductase, that play central roles in detoxifying reactive oxygen species [14,17]. During the transition period, however, the free-radical load in cows surges as a direct consequence of an increased metabolic rate, accelerated lipolysis, and the onset of lactation. The problem is further exacerbated by an often-suboptimal dietary intake of selenium in certain geographic regions where soils have a low Se content. The net effect is that oxidative stress can compromise immune function, metabolic homeostasis, and reproductive processes [7]. Selenium, as an integral component of selenoproteins, is critical for mitigating these processes, yet conventional supplementation approaches—like inorganic sodium selenite—have certain limitations, including less efficient absorption or slower kinetics of tissue assimilation. Conversely, organic forms, such as selenium yeasts or selenomethionine, can be better utilized, although high-dose usage may raise concerns about toxicity or interactions with other dietary components [13].

Selenitetriglycerides, derived from the chemical modification of sunflower oil with selenic acid [23,24], represent a more recently explored organic selenium form. Their lipophilic nature significantly enhances their absorption in ruminants [25]. Multiple investigations have revealed that selenitetriglycerides produce a rapid and pronounced increase in serum Se concentrations, with minimal risk of toxicity when used prudently [25,26]. Our work corroborates these findings. We observed that orally administered selenitetriglycerides quickly elevated selenium concentrations in postpartum cows, suggesting that these compounds can help correct shortfalls in Se status during the period of highest antioxidant demand. Because the postpartum interval is typified by a high metabolic rate and considerable immunologic shifts, an expedient method of replenishing Se reserves has important implications for disease control and overall performance [13].

Our results point toward improvements in antioxidant capacity in the supplemented group, as indicated by increased GSH-Px activity. Mechanistically, by supporting GSH-Px, selenium interrupts the cycle in which reactive oxygen species aggravate inflammation and hamper insulin sensitivity [14]. The interplay between oxidative stress and inflammation in cows is well documented: an upregulation of inflammatory cytokines—such as tumor necrosis factor alpha (TNF-α)—impairs hepatic lipid metabolism and fosters insulin resistance in adipose tissue, thus fueling the NEB that arises around calving [5,6]. In a scenario where antioxidant defenses become inadequate, these inflammatory cues intensify, establishing a vicious cycle. Our data imply that selenitetriglycerides bolster the organism’s capability to terminate this destructive feedback loop by reinforcing enzymatic antioxidants and mitigating excessive TNF-α release.

Beyond its direct antioxidant role, selenium has been shown to modulate the expression of specific genes, notably those associated with inflammatory and metabolic regulation [5,27,28]. In dairy cows, peroxisome proliferator-activated receptors (PPARs) hold a central position in regulating NEFA oxidation, gluconeogenesis, and energy partitioning [21]. Within this family, PPAR-α is prominent in hepatocytes, where it facilitates the transcription of β-oxidation enzymes, ketogenesis, and partial anti-inflammatory responses [6]. At the same time, PPAR-δ plays a role in muscle fatty acid oxidation and insulin sensitivity [11]. Excessive TNF-α can downregulate these PPARs, restricting the cow’s capacity for efficient lipid metabolism and aggravating NEB. By diminishing inflammatory overshoot and ensuring robust selenoprotein activity, selenitetriglycerides potentially sustain PPAR function, thus keeping the hepatic lipid content in check and possibly lessening postpartum ketosis. Although we did not explicitly measure PPAR protein expression, assessing only mRNA expression in our experiment, the observed beneficial metabolic trends and reduced inflammatory indices in supplemented cows suggest the enhanced activity of these nuclear receptors.

Furthermore, the postpartum interval involves a variety of hepatic gene expression shifts related to nutrient metabolism, immune activity, and stress adaptation [7]. Studies employing transcriptomic methods have identified that subclinical deficits in selenium or surges in TNF-α can profoundly alter the transcript abundance of critical metabolic and immune genes [6,11]. The synchronization of these networks relies heavily on the body’s antioxidant defenses. Our findings, which show a capacity for selenitetriglycerides to raise Se levels speedily while simultaneously minimizing negative consequences of lipolysis and oxidative stress, add valuable evidence that addressing maternal Se needs before and right after calving not only decreases postpartum pathology but may also have beneficial repercussions for fertility and subsequent lactation performance.

Inflammatory conditions such as metritis or endometritis remain principal reasons for fertility challenges in dairy herds [2,8]. These pathologies are often accompanied by elevated TNF-α, IL-1, IL-6, or other cytokine levels, which can derail ovarian follicle dynamics, corpus luteum maintenance, and embryo survival [29,30,31]. By fortifying antioxidant defenses, selenitetriglycerides might preempt an overproduction of reactive oxygen species that otherwise trigger or amplify the cytokine surge. This protective effect can help to maintain a more homeostatic uterine environment, thereby fostering normal ovarian function. Although our data did not directly evaluate reproductive endpoints, the mechanistic link between improved Se status, dampened inflammation, and fertility has been widely documented [15,32,33]. Consequently, selenitetriglycerides hold promise not just for controlling postpartum metabolic issues but also for supporting subsequent reproductive success.

The issue of selenium toxicity arises if supplementation exceeds recommended doses. Chronic over-supplementation can cause selenosis, manifesting in clinical signs from hair loss to neurological disturbances. Some inorganic Se forms or inappropriate feed blending can elevate this risk [17,23]. However, selenitetriglycerides exhibit comparatively low toxicity, presumably because of their controlled absorption and metabolism, in addition to the stable bonding between Se and fatty acid moieties [24]. Our previously published studies confirm no detectable sign of hepatic or renal impairment in cows receiving oral selenitetriglycerides at 0.5 mg Se per kg body weight. Coupled with the speed of absorption, that fosters a favorable safety and efficacy profile for periparturient use [13]. Nevertheless, verifying adherence to best practices in terms of dosage and verifying diet composition remain indispensable for preventing inadvertent overdose.

Finally, it is worth hypothesizing that some of the beneficial impacts of selenitetriglycerides could be mediated through epigenetic mechanisms. Past research has indicated that selenium compounds can influence epigenetic marks such as DNA methylation and histone modifications [34,35]. Given selenium’s role in maintaining the intracellular redox balance, it is plausible that selenitetriglycerides could indirectly modulate the epigenetic regulation of key inflammatory genes chronically activated during postpartum disorders [36]. However, this remains a compelling but untested hypothesis. Therefore, future studies employing multi-omics and epigenetic profiling are essential to determine whether such nuanced regulatory effects contribute to the observed benefits of supplementation.

Despite these promising findings, which raise further scientific questions and possibilities, our study exhibits several constraints that warrant consideration when interpreting the results. The relatively small number of animals, all drawn from a single herd, may limit the broader applicability of our conclusions. Additionally, relying on mRNA expression as the primary biomarker restricts mechanistic insights, as protein-level assessments of TNF, PPAR-α, and PPAR-δ would offer more definitive evidence of the observed regulatory changes. The absence of direct measurements for postpartum fertility or disease incidence further complicates the ability to establish a clear link between molecular alterations and improvements in overall health or productivity. Finally, this study encompassed only the immediate periparturient phase and did not extend into later lactation, where the more sustained effects of selenium supplementation could manifest. Future investigations with larger and more diverse cohorts, comprehensive protein quantifications, and extended follow-up periods are needed to substantiate and refine these initial observations.

## 4. Materials and Methods

### 4.1. Animals and Methodology

**Figure 2:**
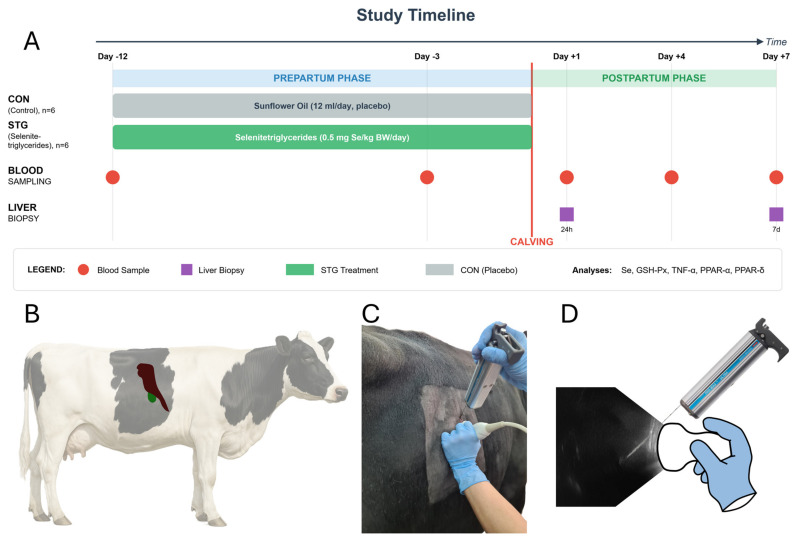
Experimental schedule for oral selenitetriglyceride supplementation and sampling in periparturient Holstein–Friesian cows. (**A**) Twelve cows were allocated to a control group (CON; n = 6) or a selenitetriglyceride-supplemented group (STG; n = 6). STG-group cows received 0.5 mg Se/kg body weight day orally from 12 days before the expected calving date (−12 d) until parturition (day 0); CON cows received an equal volume of sunflower oil on the same schedule. Venous blood samples were collected on days −12 and −3 prepartum and on days +1, +4, and +7 postpartum (red circles). Ultrasound-guided liver biopsies were obtained at 24 h (+1 d) and 7 d after calving (purple squares). Serum selenium concentration, erythrocyte glutathione peroxidase (GSH-Px) activity, and hepatic mRNA abundance of *TNF-α*, *PPAR-α*, and *PPAR-δ* were quantified in the respective samples. (**B**) Right-lateral silhouette of a Holstein–Friesian cow showing the acoustic window over intercostal spaces 9–11, which provides sonographic access to the right liver lobe (dark red)—the region available for biopsy and gallbladder (green). (**C**) In-procedure photograph: a convex transducer is positioned between the 10th and 11th intercostal spaces while a spring-loaded biopsy gun fitted with a disposable needle (1.6 mm diameter, 20 cm length) is advanced through tissues under real-time ultrasound guidance. The operator’s left hand stabilizes the probe; the right hand activates the biopsy device. (**D**) Schematic composite of the ultrasonogram and instrument orientation. The hyperechoic biopsy needle (bright line) is seen progressing through the ultrasound beam toward hepatic tissue, with overlaid outlines of the probe (white) and biopsy gun (Pro-Mag Ultra, Argon Medical Devices) illustrating coordinated manipulation.

The present study was performed as an extension of our earlier investigation on selenitetriglyceride supplementation in dairy cows [13]. In adherence to the 3R principle (Replacement, Reduction, and Refinement), which aims to minimize additional animal use, leftover serum and liver samples from the prior experiment were repurposed to explore a new research hypothesis. Briefly, the initial trial involved twelve multiparous Holstein–Friesian cows, all in their third pregnancy, selected from a single commercial dairy herd in northeastern Poland. The cows were managed in a free-stall housing system with deep bedding, featuring natural light from skylights and unrestricted access to an outdoor paddock. Water was available ad libitum, and the herd were milked twice daily. To establish comparable experimental groups, the selected cows were evenly allocated into a control (CON; n = 6) and a treatment (STG; n = 6) group, matched for parity and body condition score (BCS 3.5–3.75). The study was conducted from January through April to prevent heat stress from acting as a confounding variable. Aside from the experimental supplementation, all housing, management, and dietary conditions were identical for both groups throughout the trial. The experimental (STG) group received a daily oral dose of selenitetriglycerides at 0.5 mg Se per kg body weight, starting 12 days before the expected calving date and continuing until parturition. The control group (CON) were given a placebo of 12 mL of sunflower oil on the same schedule. The experimental timeline of supplementation and sampling is illustrated in Figure 2.

Detailed information on the feeding programs for the dry period and early lactation period is available in the original publication [13]. The cows were fed a Total Mixed Ration (TMR). The ration for the dry period was formulated to meet a target dry matter intake (DMI) of approximately 2% of body weight (e.g., for a 600 kg cow, 2% DMI equals 12 kg), resulting in a daily provision of 11.72 kg DMI per cow. The early lactation ration was formulated to provide 25.10 kg of DMI per cow. The rations included a comprehensive vitamin–mineral complex to support the metabolic demands of the transition period. The complex, including vitamins A (225,000 IU/kg), D3 (60,000 IU/kg), and E (2000 mg/kg), along with multiple B vitamins, various macro- and microminerals (iron, manganese, zinc, copper, iodine, selenium in sodium selenite and selenomethionine forms, and cobalt),were provided in both stages of feeding to support the increasing metabolic demands around calving.

### 4.2. Blood Collection and Analyses

Blood was sampled five times in each cow: day −12 and day −3 pre-calving, plus day +1, day +4, and day +7 postpartum. Samples were taken from the median caudal vein two hours after morning feeding using a vacuum-based collection system and 20G 0.9 × 38 mm needles (BD Vacutainer, Becton Dickinson, Franklin Lakes, NJ, USA). Tubes with clot activator (9 mL, Vacuette, Greiner Bio-One, Les Ulis, France) were used for serum, while K2EDTA tubes (4 mL, Vacuette, Greiner Bio-One, Les Ulis, France) were employed for whole blood. Centrifugation was performed at 1500 rpm for 15 min, after which serum was frozen at −80 °C. Selenium measurements followed the hydride generation-flame atomic absorption spectrometry protocol (Unicam 939 Solar Spectrophotometer, Labexchange-Die Laborgerätebörse GmbH, Burladingen, Germany). The erythrocyte fraction was also frozen at −80 °C for subsequent tests, including the determination of glutathione peroxidase (GSH-Px) activity. Erythrocyte GSH-Px levels were assessed using an automated biochemical analyzer (ACCENT 200, Cormay, Łomianki, Poland) (Figure 2).

### 4.3. Liver Sampling and RNA Techniques

Liver biopsies were obtained at 24 h and 7 days postpartum, using ultrasound (5 MHz convex probe, 4 Vet Slim, Dramiński, Olsztyn, Poland) to localize the intercostal spaces 9 to 11. A sterile biopsy needle (1.6 mm diameter, 20 cm length, Pro-Mag Ultra, Argon Medical Devices, Athens, TX, USA) and an automatic biopsy instrument (Pro-Mag Ultra, Argon Medical Devices, Athens, TX, USA) were used to collect tissue samples from each animal at two separate time points. Local anesthesia (Polo-cainum Hydrochloricum 5% cum Adrenalino 0.005%, Biowet-Drwalew, Góra, Poland) was administered about two hours after the morning feeding (Figure 2). Samples were placed in sterile Eppendorf Safe-Lock Tubes (Eppendorf SE, Hamburg, Germany), which were then transferred in liquid nitrogen and stored at −80 °C. Tissue fragments were homogenized (TissueLyser LT, Qiagen, Hilden, Germany) using 5 mm stainless-steel beads. Total RNA was extracted with the Total RNA Mini Plus Kit (A&A Biotechnology, Gdynia, Poland), then digested with DNase, purified, and concentrated (Clean-Up RNA Concentrator Kit, A&A Biotechnology, Gdynia, Poland). RNA concentration, purity, and integrity were checked (NanoDrop ND1000 spectrophotometer, Thermo Scientific, Waltham, MA, USA, and Agilent 2100 Bioanalyzer with the RNA 6000 Nano Kit, Agilent Technologies, Santa Clara, CA, USA). The samples were then stored at −80 °C until cDNA synthesis (Figure 2).

### 4.4. cDNA Synthesis and RT-qPCR Details

One microgram of total RNA primed with oligo(dT)18 was converted into cDNA using the Maxima First Strand cDNA Synthesis Kit combined with dsDNase, following Thermo Scientific’s instructions. The resulting cDNA was diluted one hundred times and stored at −20 °C in aliquots. Quantitative PCR assays were conducted on a LightCycler LC 480 II (Roche) using the LightCycler 480 SYBR Green I Master Reagent (Roche, Vienna, Austria), and primer sequences are listed in Table 1. The amplification program included a 5 min pre-incubation at 95 °C, followed by 45 cycles of denaturation (95 °C for 10 s), primer-specific annealing temperatures (10 s), and extension (72 °C for 10 s). A subsequent melting curve was used to verify a single amplicon without primer–dimer formation, starting at 95 °C for 5 s, cooling to 65 °C for 1 min, and gradually raising the temperature from 65 °C to 97 °C at 0.11 °C/s while recording fluorescence. GAPDH and RPL32 were the reference genes for RT-qPCR data normalization. PCR efficiency (E) and error were computed through standard curves generated by diluting pooled cDNA fourfold over five measurement points. Each assay was run in duplicate. Specificity was confirmed by running 3% agarose gels. The second derivative maximum method in the LightCycler 480 SW 1.5 software determined the quantification cycle (Cq). The relative expression levels used the 2^−ΔΔCt^ approach, as outlined by Schmittgen and Livak 2008 [37].

### 4.5. The cDNA Synthesis and RT-qPCR Details

Data from text files and spreadsheets were compiled and processed with Python 3.10.0 (van Rossum and Drake, 2009) and R 4.3.1 (R Core Team, 2020). Descriptive statistics including means and standard deviations were generated for each experimental parameter at each sample date. The Shapiro–Wilk test verified normality, and Levene’s test checked the homogeneity of variances. Given the sample size and the departures from normality, non-parametric tests were selected. For pairwise comparisons on each sampling day, the Mann–Whitney U test was used. Repeated Measures ANOVA was performed to identify changes over time, applying Mauchly’s sphericity test and Greenhouse–Geisser corrections where required. Spearman’s rank correlation coefficient was calculated for gene expression relationships. Data visualization employed Matplotlib 3.10 for line graphs and bar charts. Statistical significance was declared at *p* < 0.05.

## 5. Conclusions

Our results indicate that supplementing periparturient dairy cows with selenitetriglycerides effectively elevated blood selenium levels and reinforced the antioxidative defense system, notably through higher GSH-Px activity. This enhanced antioxidant status appeared to restrict excessive TNF expression and stabilize key metabolic pathways, as evidenced by relatively unchanged PPAR-α expression and a drop in PPAR-δ expression. Consequently, selenitetriglycerides may help dairy cows to better endure the heightened metabolic and immunological stresses of early lactation, which is likely to mitigate the risk of inflammatory-driven disorders and support more efficient hepatic lipid handling.

## Figures and Tables

**Table 1:** The quantitative reverse transcription PCR (qRT-PCR) primer sequences applied in the study and the amplicons’ details.

Gene Symbol	Primer Sequences	Annealing Temp	T_m_	Amplicon Size	E	*Error*
	(F-forward/R-reverse) (5′-3′)	(°C)	(°C)	bp	10^−1/slope^	MSE
*TNF*	F—CTGGTTCAGACACTCAGGTCC R—GAGGTAAAGCCCGTCAGCA	58	87.7	183	1.99	0.005
*PPAR-α*	F—GGATGTCCCATAACGCGATT R—GGTCATGCTCACACGTAAGGATT	60	79.3	90	1.97	0.011
*PPAR-δ*	F—TGTGGCAGCCTCAATATGGA R—GACGGAAGAAGCCCTTGCA	60	86.6	100	1.99	0.007
*GAPDH* reference gene	F—GTCTTCACTACCATGGAGAAGG R—TCATGGATGACCTTGGCCAG	60	86.1	197	2.03	0.011
*RPL32* reference gene	F—AAAGAGGACCAAGAAGTTCATTAG R—CGCCAGTTCCGCTTGATTT	60	78.1	66	1.98	0.013

E, PCR efficiency calculated using formula E = 10^−1/slope^; error value, mean squared error of the single data point fit to the regression line; T_m_, melting-temperature-curve peak value.

## Data Availability

None of the data were deposited in an official repository. All of the data obtained in the present research are presented in this manuscript. The data that support the study findings are available from the authors upon request.
